# Disseminated Abdominal Hydatidosis: A Rare Presentation of Common Infectious Disease

**DOI:** 10.1155/2014/164787

**Published:** 2014-07-08

**Authors:** Abdulrahman Almalik, Aynaa Alsharidi, Mohammed Al-Sheef, Mushirah Enani

**Affiliations:** ^1^Academic and Training Affairs, King Fahad Medical City, P.O. Box 59046, Riyadh 11525, Saudi Arabia; ^2^Department of Medicine, King Khalid University Hospital, Riyadh, Saudi Arabia

## Abstract

Hydatid disease is one of the most geographically widespread zoonoses with substantial disease burden. In this report we are discussing an unusual case of intra-abdominal HD that was ongoing for 22 years despite two surgical interventions. Significant symptomatic relief was achieved within the first two months of combination therapy with albendazole and praziquantel. HD is still of public health concern in the Middle East that needs optimized care.

## 1. Introduction

Hydatid disease (HD) also known as cystic echinococcosis is a zoonotic infection caused by the cestode tapeworm* Echinococcus granulosus* and rarely by* Echinococcus multilocularis*. HD is endemic in the cattle grazing areas particularly Australia, New-Zealand, Middle East, India, Africa, South America, and Turkey. In humans the HD commonly involves the liver (75%) and the lungs (15%). The remaining (10–15%) of the cases includes the other regions of the body. Peritoneal hydatidosis is a rare presentation reported in only 2–12% of all abdominal HD.

## 2. Case Report

A 70-year-old lady came to our clinic with 2-week history of progressive abdominal pain and distension. Her problem started 22 years ago when she started to have progressive abdominal distension associated with on/off epigastric pain, dull in nature, not radiating, and associated with constipation but no nausea nor vomiting. She had low appetite, increasing fatigue, and undocumented weight loss but no fever or night sweats. She had no respiratory, cardiovascular, or genitourinary complaints. She gave a history of contact with animals but no raw milk ingestion. She has been evaluated in Kuwait and explorative laparotomy revealed intra-abdominal abscess which had been evacuated on the spot. After the operation she was hospitalized for approximately 18 months and then discharged on unknown oral medication. Three years later, she started to have progression of her symptoms and laparotomy was done for the second time.

**Figure 1:**
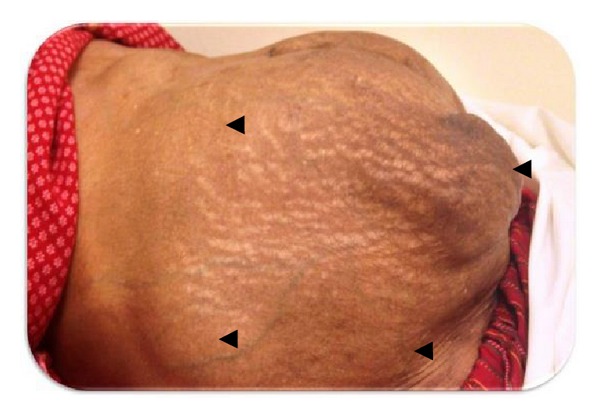
Multiple intra-abdominal masses as shown by arrow heads.

On examination she looked well, not in pain or in distress; vital signs were as follows: pulse: 77 beat/minute, blood pressure: 150/90 mmHg, respiratory rate: 20/minute, SpO_2_: 98% in room air, and temperature: 36.5°C. She had no lymphadenopathy, pallor, cyanosis, or jaundice. Abdominal examination revealed markedly distended abdomen with multiple firm intra-abdominal masses in the epigastrium, both lumbar regions and right lower quadrant, with tenderness in epigastric and left lumbar regions. There was no guarding, rigidity, or rebound tenderness (Figure 1). Cardiovascular, respiratory, nervous system, and musculoskeletal examination were unremarkable.

**Figure 2:**
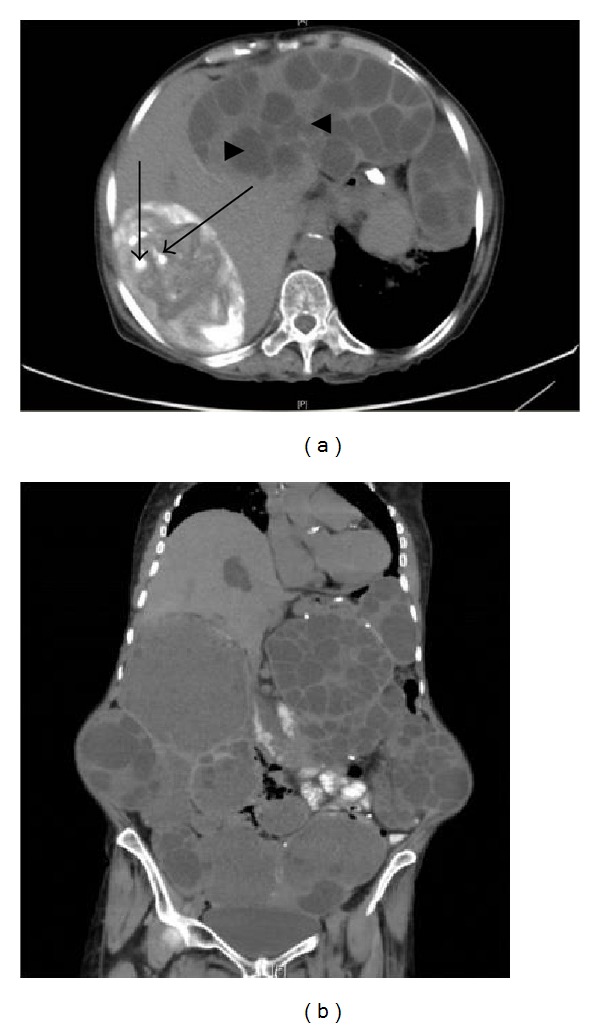
(a) CT scan abdomen showing multiple cystic lesions of variable appearance and size, with partial calcification “long arrows” and intrahepatic daughter cyst “arrow head.” (b) Multiple cystic lesions of variable appearance and size with septa and wall trabeculation.

Laboratory tests showed the following: WBC: 7.75 × 10^9^/L (4–10 × 10^9^/L), absolute eosinophil count: 1.27 × 10^9^/L (0.04−0.45 × 10^9^/L), sedimentation rate of 120 mm/H (<40 mm/H) and C-reactive protein of 38 mg/L (<6 mg/L), urea: 16.7 mmol/L (3–7 mmol/L), creatinine: 223 *μ*mol/L (60–118 *μ*mol/L), AST: 28 U/L (6–34 U/L), ALT: 20 U/L (5–21 U/L), alkaline phosphatase: 97 U/L (42–98 U/L), total bilirubin: 7.2 *μ*mol/L (2–17 *μ*mol/L), and direct bilirubin: 4.2 *μ*mol/L (0–5 *μ*mol/L). Nonenhanced abdominal computed tomography (CT) revealed multiple cystic lesions of variable appearance and size with internal membranes and daughter cysts in liver, peritoneum, spleen, and kidney, some of which are partially calcified (Figures 2(a) and 2(b)).

## 3. Discussion

Peritoneal hydatidosis is an uncommon entity of HD. Secondary peritoneal disease, which is the most common form of peritoneal HD, occurs as a result of traumatic or surgical rupture of a hepatic, splenic, or mesenteric cyst. However, spontaneous rupture of intra-abdominal hydatid microcysts into the peritoneum may also occur in about 12% of the cases. Primary hydatidosis is an extremely rare entity accounting for just 2% of all intra-abdominal hydatid disease [1–3].

Most patients remain asymptomatic for years before presenting with vague abdominal symptoms such as nonspecific pain, abdominal fullness, dyspepsia, anorexia, and vomiting [3].

Symptoms due to peritoneal hydatidosis arise commonly from complications due to enlarging abdominal cysts or rupture into the peritoneum which may present as acute abdominal pain. Antigenic fluid released into the peritoneal cavity and absorbed into the circulation may present with acute allergic manifestations. However, vague abdominal pain is the most common clinical feature [4].

The most useful diagnostic utility would be abdominal ultrasound or CT scan in which lesions appear well defined with or without internal separation [5].

In 2003, the World Health Organization Informal Working Group on Echinococcosis (WHO-IWGE) proposed a standardized ultrasound classification based on the active-transitional-inactive status of the cyst as suggested by its sonographic appearance. This classification has important implications for clinical decision-making and prognosis. Cystic echinococcosis 1 (CE1) and CE2 are active cysts containing viable protoscolices. CE3 has been subdivided into CE3a (detached endocyst) and CE3b (predominantly solid with daughter cysts). This subdivision is supported by a recent work that used high-field 1 H: magnetic resonance spectroscopy to evaluate ex vivo the metabolic profiles of cyst contents. CE 4 and CE 5 are inactive cysts which have normally lost their fertility and are degenerative. In contrary to what was previously assumed, W. Hosch and his colleagues has shown that calcification of the cyst is not restricted to the inactive WHO cyst types CE4 and CE5 but occurs in all stages and in up to 50% of cysts [6].

There are several serological tests which can be used to diagnose HD, but they have a significant difference in terms of sensitivity and specificity. Detection of antibodies has a higher sensitivity than detection of antigens [7].

The treatment of choice for localized hydatid cysts in liver or lungs is principally surgical while therapy for disseminated peritoneal hydatidosis remains medical [8].

The combination of albendazole and praziquantel has been investigated in vivo in a rat model of hydatid infection. In contrast to monotherapy with either agent, combination treatment produced a significant reduction in both the number and viability of cysts [9, 10]. Albendazole is rapidly converted to an active metabolite, albendazole sulfoxide, which achieves high concentrations in the cyst and is active against both protoscolices and the germinal membranes [11]. Praziquantel does not penetrate into the mature cyst and, therefore, does not inhibit cyst growth, but it is a highly effective protoscolicidal agent both in vitro and in vivo [12, 13]. The likely role for praziquantel in human hydatidosis may be in preventing encystment of protoscolices following perioperative spillage [12].

Todorov and his colleagues evaluated the degenerative changes seen in the cyst with benzimidazole therapy in 122 patients and they found that the initial change to be seen in abdominal cysts is detachment of the endocyst followed by hyperdense appearance and shrinking of the cyst until it finally disappears [14]. This process may take up to one year of treatment and it is indicative of the parasiticidal effect of benzimidazoles on the cysts [14].

Our patient mostly developed disseminated peritoneal hydatidosis either secondary to rupture of the primary lesion in the liver or spillage from her previous surgeries. She was started on combination medical therapy with albendazole 400 mg twice daily and praziquantel 600 mg three times daily in an outpatient setting with follow-up evaluation by imaging for signs of inactivity 6 months after chemotherapy initiation. Interestingly she started to have symptomatic relief within the first 2 months of the planned 12-month course.

## 4. Conclusion

Disseminated abdominal hydatidosis is uncommon presentation of a common zoonosis. The slowly progressive nature of the diseases explains chronicity of symptoms that may last for decades as in our patient. Combination treatment with albendazole and praziquantel has shown superiority to monotherapy.
